# Mechanistic models of humoral kinetics following COVID-19 vaccination

**DOI:** 10.1098/rsif.2024.0445

**Published:** 2025-01-29

**Authors:** Daniel Stocks, Amy Thomas, Adam Finn, Leon Danon, Ellen Brooks-Pollock

**Affiliations:** ^1^ School of Engineering Mathematics and Technology, University of Bristol, Tankard’s Close, Bristol, BS8 1TW, UK; ^2^ Population Health Sciences, Bristol Medical School, University of Bristol, Oakfield Grove, Bristol, BS8 2BN, UK

**Keywords:** within-host model, SARS-CoV-2, vaccination, antibody response, short- and long-lived plasma cell kinetics

## Abstract

COVID-19 vaccine programmes must account for variable immune responses and waning protection. Existing descriptions of antibody responses to COVID-19 vaccination convey limited information about the mechanisms of antibody production and maintenance. We describe antibody dynamics after COVID-19 vaccination with two biologically motivated mathematical models. We fit the models using Markov chain Monte Carlo to seroprevalence data from 14 602 uninfected individuals in England between May 2020 and September 2022. We analyse the effect of age, vaccine type, number of doses and the interval between doses on antibody production and longevity. We find evidence that individuals over 35 years old twice vaccinated with ChAdOx1-S generate a persistent antibody response suggestive of long-lived plasma cell induction. We also find that plasmablast productive capacity is greater in: younger people than older people (≤4.5-fold change in point estimates); people vaccinated with two doses than one dose (≤12-fold change); and people vaccinated with BNT162b2 than ChAdOx1-S (≤440-fold change). We find the half-life of an antibody to be 23–106 days. Routinely collected seroprevalence data are invaluable for characterizing within-host mechanisms of antibody production and persistence. Extended sampling and linking seroprevalence data to outcomes would enable conclusions about how humoral kinetics protect against disease.

## 1. Introduction

Vaccination against COVID-19 is now routinely used to maintain levels of population immunity. The antibody response to vaccination varies with age [1–4], peaking within a couple of weeks following vaccination and subsequently waning over the course of six months [4–6]. The mRNA Pfizer-BioNTech vaccine (BNT162b2) elicits a stronger antibody response than the adenoviral vector AstraZeneca vaccine (ChAdOx1-S) after the first dose and up to two weeks after the second dose [7], with the response to the second dose being more than an order of magnitude greater than the response to the first dose [7–10]. Characterizing antibody dynamics following vaccination is a consideration in designing vaccine programmes that maintain high levels of population immunity.

Mathematical models are used to characterize the underlying mechanisms of the immune response, explain differences between age and vaccines and predict future antibody levels. Previous modelling of SARS-CoV-2 specific antibody levels often used statistical, single-phase exponential decay models implemented through linear regression [11–16]. Such exponential decay models showed the rate of overall antibody level decline. However, these models convey limited mechanistic information because they may not be biologically motivated and are constrained by the inherent assumptions in their structure. More flexible dual-phase models [17] can begin to address these limitations, but mechanistic models can be more informative about the process of antibody maintenance, though they can suffer from identifiability issues [18,19].

Analysis of the immune response is challenging because there are many unobserved components. Quantification of serum antibody concentrations offers a viable window into the dynamics of the humoral (antibody producing) system [20–22]. Serum antibody concentrations can be quantified by taking blood samples. Plasma cells, that reside in the bone marrow [23–26], and their precursor B cells, which differentiate in lymph nodes, are much more difficult to measure without invasive procedures. It is therefore of interest to see whether information about the underlying kinetics of antibody production can be extracted from data on antibody levels alone.

### 1.1. Maintenance of a persistent immune response

Biological mechanisms that maintain the humoral response after exposure (natural infection or vaccination) have been investigated [27]. It has been found that hypotheses of persisting antibody levels as a result of memory B cells being continuously stimulated through chronic infection, re-exposure, persistent antigen or bystander T cells are not consistent with observations. The re-exposure and bystander T-cell hypotheses [27] rely on pathogens being endemic and therefore cannot explain persisting antibodies in the absence of local outbreaks [28–30]. The persistent antigen hypothesis is based on evidence that follicular dendritic cells (FDCs) sequester antigen in the later stages of infection and, over time, display it to memory B cells, encouraging a continued humoral immune response in the absence of infection [31–33]. However, the decay of antigen retained by FDCs in mice is too fast to explain stable antibody production [34]. Although persistent antigen could be important early in the memory response.

Other hypotheses explain persistent antibody levels with independently regulated plasma cells, instead of memory B cells. It has been shown that plasma cell populations and antibody levels persist in the absence of memory B cells [28,35–39]. One hypothesis that links plasma cell populations to antibody levels is competition between newly produced plasmablasts and residing, long-lived plasma cells for niches in the bone marrow [23–26,40]. However, this hypothesis predicts a faster decline in long-term antibody levels in older people, which is not observed [28]. A different hypothesis (the ‘*imprinted lifespan*’ hypothesis) proposed in [27] is that plasma cells are imprinted with a short or a long lifespan upon differentiation from B cells. B cells that differentiate without interaction with CD4${,}^{+}$ T cells (T-cell independent) and are cross-linked by repetitive foreign antigens become short-lived plasma cells (plasmablasts) [27]. Whereas a lifespan of years or decades is thought to be achieved by B cells that are cross-linked by a non-repetitive foreign antigen and interact with CD4${,}^{+}$ T cell (T-cell dependent) [27]. To acquire even longer lifespans (potentially as long as the person), B cells must be cross-linked by repetitive foreign antigens and interact with CD4${,}^{+}$ T cells [27].

In this article, we build on the imprinted lifespan hypothesis [21] to develop two mathematical models of antibody dynamics following COVID-19 vaccination. We use data from a seroprevalence survey of residual samples from primary care in England to estimate key immunological parameters. We aim to develop a modelling framework for gaining insight into the mechanisms of antibody maintenance post-vaccination.

## 2. Method and materials

**Figure 1.**
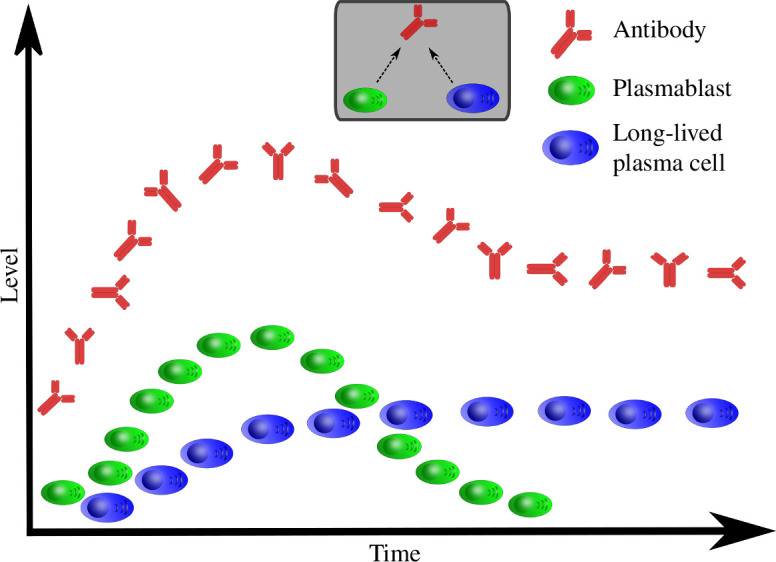
A schematic of modelled humoral dynamics. A persistent antibody response is dependent on the generation of long-lived plasma cells. In contrast, early antibody dynamics and peak serum antibody concentrations are driven by short-lived plasma cells (plasmablasts).

Following a mathematical formulation of the imprinted lifespan hypothesis proposed in [21], we analyse two mathematical models. One model includes long-lived plasma cells, and the other does not. Figure 1 shows a qualitative description of the modelled humoral dynamics, similar to those observed *in vivo* [41]. We impose the requirement that models are structurally identifiable and then ensure that they are practically identifiable, i.e. that observed outputs can only be generated by a single parameter set (a different parameter set would generate a different output) [42–44], and that the data contain enough information to inform the parameters.

We assess parameter identifiability to understand which model describes the kinetics of the humoral response after one and two doses of COVID-19 vaccination for different ages and vaccine schedules. From our analysis, we draw conclusions on which groups show evidence of long-term antibody responses, how age and vaccine schedule impact antibody levels, and the importance of a second dose.

### 2.1. Antibody data

**Figure 2.**
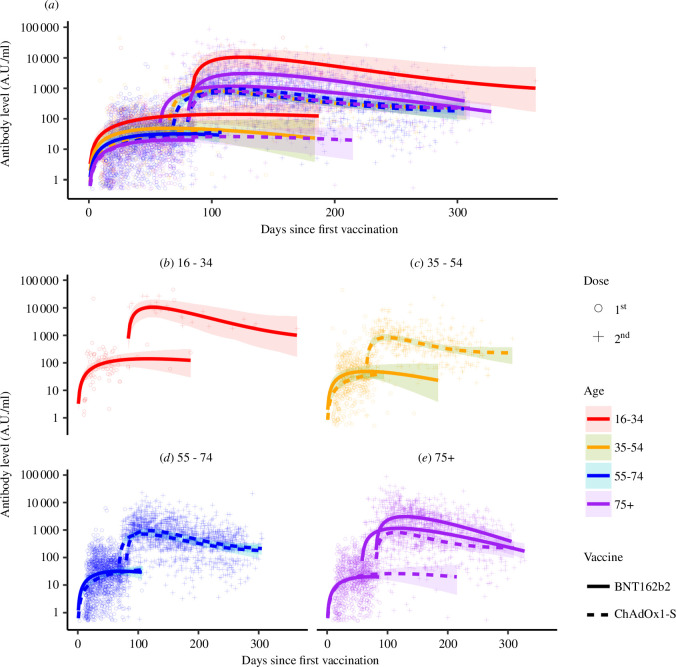
The antibody data and best fitting model of the groups whose data could inform a model (the groups in table 2). The models for people who have received two doses of ChAdOx1-S are the asymptotic model, and all other models are the single cell model. For the first dose, we initialize the models at zero ($A_{0}=0$). All parameter estimates are given in table 2. The shaded regions are the 95% HPD CrI interval of the mean, defined by the 95% HPD CrI of the parameter estimates. All identifiable model fits are plotted in (*a*) and the subsequent panels (*b*–*e*) are the same fits split by age group. There are two purple solid lines (75+ year olds who have received two doses of BNT162b2) and two dashed blue lines (55–74 who have received two doses of ChAdOx1-S) because those vaccinated ≤75 days and >75 days apart could both inform the models. For separate plots of individual group fits, see electronic supplementary material.

We use SARS-CoV-2 specific antibody data collected from 3 55 019 routine blood tests between May 2020 and September 2022 collected and validated by the Oxford-Royal College of General Practitioners (RCGP) (see figure 2) [45]. This dataset contains vaccination dates and level of total antibody (IgG, IgA and IgM) specific to the SARS-CoV-2 receptor binding domain (RBD) on the spike (S) protein [46] and nucleoprotein (N) [46]. Antibody was measured using a commercially available assay in a sandwich ELISA format (Roche Elecsys(R) spike and nucleoprotein assays). Assays are semi-quantitative with results expressed as arbitrary units per millilitre (AU ml^−1^), following interpolation to an internal control standard curve. Data are collected at various times after one, two, three or four doses. Each sample is collected from a different individual. Due to the sparsity of data after three and four doses, we only use the data after one and two doses.

While acknowledging the imperfect nature of the method, we distinguish previous infection with SARS-CoV-2 from vaccination by assessing whether an individual’s antibodies bind to N as well as S protein. Response to both proteins has been recorded in the dataset alongside an individual’s vaccine type, number of doses, interval between doses and age. These factors are used to stratify the population into groups. We assume that the antibody dynamics of the people within a group are the same; therefore, individuals’ one-off samples can be interpreted as longitudinal data for the group. As we are investigating the effects of vaccination on antibody levels, we remove anyone who has evidence of previous infection, i.e. antibodies that bind to the N protein (N positive).

Individuals are categorized by age: 16–34, 35–54, 55–74 and 75+ years. We included data on one and two doses of BNT162b2 and ChAdOx1-S vaccines, and whether the doses were administered less than or equal to 77 days apart or more than 77 days apart (reflective of the two dose intervals used in the UK). As we have set four age categories, two vaccine categories and two dose interval categories, there are eight possible groups for the first dose and 32 possible groups for the second dose. We have the minimum necessary data (i.e. as many time points as parameters for our largest model) for all first-dose groups but only 16 of the second-dose groups. We do not have the minimum necessary data for people who were heterologously vaccinated.

### 2.2. Mathematical models

The mechanistic mathematical model we propose for describing antibody dynamics post-vaccination is


(2.1) $$A(t)=\frac{\Phi_{l}}{\mu_{a}}+\frac{\Phi_{s}}{\mu_{a}-\mu_{s}}e^{-\mu_{s}t}+\left(A_{0}-\frac{\Phi_{l}}{\mu_{a}}-\frac{\Phi_{s}}{\mu_{a}-\mu_{s}}\right)e^{-\mu_{a}t},$$

where $\Phi_{s}$ and $\Phi_{l}$ represent the maximum antibody production of short-lived and long-lived plasma cells respectively, and $\mu_{a}$ and $\mu_{s}$ are decay rates of antibody and short-lived plasma cells respectively. We will refer to $\Phi_{s}$ and $\Phi_{l}$ as the productive capacity. It is assumed long-lived plasma cells do not decay. For derivation see appendix A.

We also consider a related model (the single-cell model) that assumes long-lived plasma cells are not induced ($\Phi_{l}=0$).


(2.2) $$A(t)=\frac{\Phi_{s}}{\mu_{a}-\mu_{s}}\left(e^{-\mu_{s}t}-e^{-\mu_{a}t}\right)+A_{0}e^{-\mu_{a}t}.$$

This distinction is made to improve the identifiability of the model by removing uncertainty (see appendix B).

When describing antibody dynamics after the first dose, it is assumed $A_{0}=0$ in both models.

### 2.3. Model fitting

We use a Bayesian framework to fit both models (equations (A 2) and (A 3)) to data from all age groups, vaccine types, dose intervals and the number of doses, implemented in R with the rstan package (available at: https://mc-stan.org/). A Markov chain Monte Carlo (MCMC) algorithm is used to estimate the joint posterior distribution of the parameters.

To obtain a likelihood function, we assume that the log of the antibody levels can be modelled as a random variable, X(t), drawn from a normal distribution with a mean defined by our model, A(t,θ), and a standard deviation σ.


$$\begin{array}{rl} X(t) & ∼N(A(t,\theta ),\sigma ),\\ P(X(t)=x|\theta ) & =\frac{1}{\sigma \sqrt{2\pi }}e^{-\frac{(x-A(t,\theta ){)}^{2}}{2\sigma^{2}}}. \end{array}$$

#### 2.3.1. Initial conditions and prior distributions

For the first dose response, we set the initial antibody level, $A_{0}$, to zero (as we assume people have not been exposed to SARS-CoV-2), and for the response to the second dose, we assume $A_{0}$ is log-normally distributed with mean $\mu_{A_{0}}$ and standard deviation $\sigma_{A_{0}}$. The values of $\mu_{A_{0}}$ and $\sigma_{A_{0}}$ are estimated from the antibody levels of people recorded after their first dose. As the data are cross-sectional, we cannot know what each individual’s antibody level was when they were given their second dose. Hence, we sample the log-transformed serum antibody concentrations after the first dose on the days when individuals received their second dose. The mean and standard deviation of this normally distributed sample are used as $\mu_{A_{0}}$ and $\sigma_{A_{0}}$ respectively.

For the decay parameters, $\mu_{s}$ and $\mu_{a}$, we use the same log-normal prior for both. A log-normal prior is used because we expect the values of $\mu_{s}$ and $\mu_{a}$ to be small [29,35,47–53], but greater than zero. We calibrate the prior by setting its 2.5% and 97.5% quantiles to 0.01 and 0.1 respectively (equivalent to assuming there is a 95% probability that the true parameter value lies between these values). This reflects the belief that the expected half-lives of antibodies and short-lived plasma cells are in the tens of days [29,35,47–53].

The production capacity parameters, $\Phi_{s}$ and $\Phi_{l}$ are more difficult to inform from literature as they are artificial. However, $\Phi_{l}$ can still be informed through assumptions about the long-term dynamics of the model. The resting antibody level at $t_{\infty }$is predicted by the asymptotic model (equation (A 2)) to be $\frac{\Phi_{l}}{\mu_{a}}$, so $\Phi_{l}$ will be equal to the product of $\mu_{a}$ and the resting antibody level, which we denote ${\text{lAb}}_{\infty }$. We have assumed that both the antibody data and $\mu_{a}$ are log-normally distributed. The product of two log-normal distributions is log-normally distributed. If we say the mean and standard deviation of the prior for $\mu_{a}$ is $\mu_{\text{decay}}$ and $\sigma_{\text{decay}}$ respectively, and the mean and standard deviation of the resting antibody level is $\mu_{\infty }$ and $\sigma_{\infty }$ respectively, the prior for $\Phi_{l}$ will have the mean and standard deviation $\mu_{\Phi_{l}}=\mu_{\text{decay}}+\mu_{\infty }$ and $\sigma_{\Phi_{l}}=\sigma_{\text{decay}}^{2}+\sigma_{\infty }^{2}-2\sigma_{\text{decay}}\sigma_{\infty }$. We calculate $\mu_{\infty }$ and $\sigma_{\infty }$ through assuming the 2.5% and 97.5% quantiles of the distribution. After the first dose, we assume the 2.5% and 97.5% quantiles of the log-transformed resting antibody level will be 2 and 7, respectively, and after the second dose, 3 and 8, respectively. These quantiles are validated by prior predictive checks and informed by the data we have on earlier time points; therefore, they do not violate the principle of prior belief being ignorant of data, as we do not have data over a long time.

The final parameter is the production capacity of short-lived plasma cells, $\Phi_{s}$. There is not the same intuitive interpretation for $\Phi_{s}$ in the dynamics of the model as there is for $\Phi_{l}$ so we use a truncated normal distribution with the 2.5% and 97.5% quantiles 1 and 5, respectively, for the first dose and 50 to 1000, respectively, for the second dose. These quantiles were calibrated through prior predictive checks.

To summarize, the prior distributions are,


(2.3a) $$\begin{array}{lrlr} & \Phi_{s} & ∼Truncnormal(\mu_{\Phi_{s}},\sigma_{\Phi_{s}}), & \end{array}$$

(2.3b) $$\begin{array}{lrlr} & \Phi_{l} & ∼Lognormal(\mu_{\Phi_{l}},\sigma_{\Phi_{l}}), & \end{array}$$

(2.3c) $$\begin{array}{lrlr} & \mu_{s} & ∼Lognormal(\mu_{\text{decay}},\sigma_{\text{decay}}), & \end{array}$$

(2.3d) $$\begin{array}{rl} \mu_{a} & ∼Lognormal(\mu_{\text{decay}},\sigma_{\text{decay}}), \end{array}$$

(2.3e) $$\begin{array}{rl} A_{0} & ∼Lognormal(\mu_{{\text{A}}_{0}},\sigma_{{\text{A}}_{0}}), \end{array}$$

#### 2.3.2. Parameter identifiability

We assess parameter identifiability by conducting a sensitivity analysis of the posterior to the priors. For the sensitivity analysis, we expand the quantiles of the prior distributions of $\Phi_{s}$, $\mu_{s}$, $\mu_{a}$, ${\text{lAb}}_{\infty }$, and double the standard deviation calculated for a particular group, shown in table 1. The quantiles of the sensitivity analysis prior distributions are chosen to widen and weaken the prior distribution. Care is taken when choosing the expanded quantiles to prevent the mode from spiking (which can happen with log-normal distributions with a small mean but large standard deviation), as this would result in a stronger rather than a weaker prior. To determine whether a 95% HPD CrI is sensitive to the priors, we check two things: whether the inclusion of zero in the HPD CrI has changed, and whether the modes of both posteriors are included within the HPD CrI of both posteriors. If the inclusion of zero in HPD CrI changes, so does the conclusion about whether the effect of the parameter can be detected in the data. This is a fundamental change, and we say the posteriors are too sensitive to the prior in this case. Checking if the modes of the posteriors are included within the HPD CrI of the other posterior is to test for agreement in the estimates. If there is agreement, we say the posterior is not too sensitive to the priors.

**Table 1.** Prior distributions for the model parameters. For $\Phi_{s}$, $\mu_{s}$, $\mu_{a}$, and ${\text{lAb}}_{\infty }$ we assume the 2.5% and 97.5% quantiles of the prior distributions. For $A_{0}$ the assumptions are the mean and standard deviation. Values are not given for the $A_{0}$ assumptions as they vary between the groups.

dose					
	baseline prior assumptions				
	$\Phi_{s}$	$\mu_{s}$	$\mu_{a}$	${\text{lAb}}_{\infty }$	$A_{0}$
$1^{\text{st}}$	[1,5]	[0.01, 0.1]	[0.01, 0.1]	[2,7]	—
$2^{\text{nd}}$	[46, 1000]	[0.01, 0.1]	[0.01, 0.1]	[3,8]	$\mu_{A_{0}}$, $\sigma_{A_{0}}$
	sensitivity analysis prior assumptions				
	$\Phi_{s}$	$\mu_{s}$	$\mu_{a}$	${\text{lAb}}_{\infty }$	$A_{0}$
$1^{\text{st}}$	[0.5, 10]	[0.005, 0.1]	[0.005, 0.1]	[1,8]	—
$2^{\text{nd}}$	[46, 2000]	[0.005, 0.1]	[0.005, 0.1]	[2,9]	$\mu_{A_{0}}$, $2\,\sigma_{A_{0}}$

## 3. Results

### 3.1. Antibody dynamics by age and vaccine type

We split the data on antibody level into age groups (16–34, 35–54, 55–74, 75+), vaccine type (BNT162b2, ChAdOx1-S), number of doses (one or two) and the interval between doses (< 77 days, ≥ 77 days). Debated intervals in the UK, prior to model fitting. This splitting produces eight groups post first vaccination and 16 groups post second vaccination. Using mathematical models of antibody dynamics (equation (A 2) and (A 3)), we are able to describe the SARS-CoV-2 specific antibody response of seven of the eight first dose groups and seven of the 16 second dose groups (see table 2). The data of all the groups together (the data are not split by dose, age, vaccine type, etc.) could not inform the models (i.e. the models were not practically identifiable with their data). The groups that could not inform the models tended to have smaller sample sizes than the groups that could.

**Table 2.** The vaccine schedule, age, size, best fitting model, parameter estimates and 95% HDP CrI of the groups whose data could identify a model. ‘Pfz' and ‘Az' are shorthand for BNT162b2 and ChAdOx1-S respectively. ‘SC’ and ‘Asym’ are shorthand for the single cell and asymptotic models respectively. The parameters $\Phi_{s}$, $\Phi_{l}$, $\mu_{s}$, $\mu_{a}$ and $A_{0}$ are the short-lived plasma cell productive capacity, the long-lived plasma cell productive capacity, the short-lived plasma cell decay rate, the antibody decay rate, and the initial level of antibody respectively.

vaccine		age	dose	sample	model	parameter estimates				
$1^{\text{st}}$	$2^{\text{nd}}$		interval	size		$\Phi_{s}$	$\Phi_{l}$	$\mu_{s}$	$\mu_{a}$	$A_{0}$
Az	—	35–54	—	352	SC	0.835	—	0.00588	0.00593	—
						[0.673–1.07]		[0.00214–0.0142]	[0.00216–0.0143]	
Az	—	55–74	—	870	SC	0.621	—	0.00490	0.00509	—
						[0.535–0.738]		[0.00193–0.0109]	[0.00191–0.0109]	
Az	—	75+	—	300	SC	0.615	—	0.00731	0.00737	—
						[0.481–0.831]		[0.00241–0.0177]	[0.00251–0.0179]	
Pfz	—	16–34	—	92	SC	3.20	—	0.00672	0.00673	—
						[2.30–4.52]		[0.00219–0.0182]	[0.00219–0.0182]	
Pfz	—	35–54	—	182	SC	1.92	—	0.0114	0.0118	—
						[1.31–2.91]		[0.00337–0.0324]	[0.00341–0.0324]	
Pfz	—	55–7 4	—	532	SC	1.20	—	0.116	0.0121	—
						[0.923–1.56]		[0.00409–0.0258]	[0.00413–0.0260]	
Pfz	—	75+	—	547	SC	0.722	—	0.0114	0.0116	—
						[0.551–0.976]		[0.00388–0.0256]	[0.00391–0.0257]	
Az	Az	35–54	>77 days	453	Asym	59.5	5.82	0.0259	0.0294	25.8
						[44.2–81.3]	[1.67–12.4]	[0.0107–0.0539]	[0.0147–0.0598]	[10.9–59.7]
Az	Az	55–74	≤77 days	967	Asym	61.8	4.85	0.0252	0.0276	12.5
						[51.5–74.5]	[1.96–8.56]	[0.0146–0.0400]	[0.0171–0.0429]	[4.45–30.7]
Az	Az	55–74	>77 days	695	Asym	43.8	3.60	0.0209	0.0267	12.9
						[34.9–55.9]	[1.20–7.43]	[0.0109–0.0400]	[0.0149–0.0479]	[3.07–41.1]
Az	Az	75+	≤77 days	285	Asym	57.9	6.16	0.0284	0.0299	24.2
						[37.9–88.3]	[1.74–13.5]	[0.0102–0.0584]	[0.0144–0.0627]	[6.79–73.2]
Pfz	Pfz	16–34	≤77 days	25	Asym	632	5.15	0.0138	0.0156	88.9
						[341–1060]	[0–40.6]	[0.00612–0.0622]	[0.00674–0.0553]	[8.64–761]
Pfz	Pfz	75+	≤77 days	430	SC	170	—	0.0164	0.0171	21.1
						[126 – 234]		[0.0100–0.0366]	[0.00992–0.0367]	[7.05–58.8]
Pfz	Pfz	75+	>77 days	661	SC	50.4	—	0.0155	0.0156	25.2
						[39.5−65.0]		[0.0102−0.0236]	[0.0102−0.0235]	[9.55−59.8]

Antibody dynamics after the first dose are best described by the single cell model. Antibody dynamics after the second dose are described best by the asymptotic model, except when people are more than 75 years old and vaccinated with two doses of BNT162b2. All first-dose groups that could inform the model could only inform the single-cell model, except for 25–54 years old vaccinated by BNT162b2. Comparing the single-cell and asymptotic models for this group by Bayes Factor, we find the marginal likelihood for the single-cell model to be greater than the asymptotic model (a Bayes Factor of 15 58 400 in favour of the single-cell model). Similarly, all second-dose groups could only inform one model except for 16–34 year-olds vaccinated with two doses of ChAdOx1-S and 55–74 year-olds vaccinated with two doses of BNT162b2. These two groups could inform both models. The marginal likelihood for the asymptotic model was greater than for the single-cell model (a Bayes factor of 12 and 63, respectively, in favour of the asymptotic model).

Figure 2 shows the dynamics of the modelled groups given in table 2 (for individual group fits see electronic supplementary material). We see that age has a greater impact on the magnitude of the antibody response for people vaccinated with BNT162b2 than those vaccinated with ChAdOx1-S. After both the first and second doses, younger people vaccinated with BNT162b2 have higher antibody levels than older people vaccinated with BNT162b2. Our models predict this trend will continue into the future. While for those vaccinated with ChAdOx1-S, there is little discrepancy between the age groups, especially after the second dose.

We also find that those vaccinated with one dose of BNT162b2 have higher antibody levels than the same age group vaccinated with one dose of ChAdOx1-S in the short term but lower levels in the long term. Younger people vaccinated with BNT162b2 have higher antibody levels than the same age group vaccinated with ChAdOx1-S for longer than older people. The time of cross-over (when antibody levels become equal) is 113.4, 90.0, and 33.4 days, for 34–35, 55–74, and 75+ year olds, respectively. However, the 95% HPD CrIs of the means overlap for the whole timeframe of the first dose data (up to 215 days post vaccination), so we cannot be confident that a cross-over occurs in the timeframe of these data. We do not have a comparison for 16–34 year-olds because the data on this age group’s response to one dose of ChAdOx1-S were insufficient to identify a model.

We also find that people vaccinated with two doses of BNT162b2 will have higher antibody levels than those vaccinated with ChAdOx1-S for approximately nine months after receiving their second dose. After nine months, the models predict those vaccinated with ChAdOx1-S will have higher antibody levels. A possible exception is 16–34 year-olds vaccinated with BNT162b2. Our analysis is uncertain about whether long-lived plasma cells are induced. The 95% HDP CrI for the group’s antibody dynamics includes decaying to zero in the long term. So, we cannot be confident that 16–34 year-olds vaccinated with two doses of BNT162b2 will have persisting antibodies. This wide interval is probably due to the small sample size of the group (n=25).

### 3.2. Estimates of humoral response parameters

**Figure 3.**
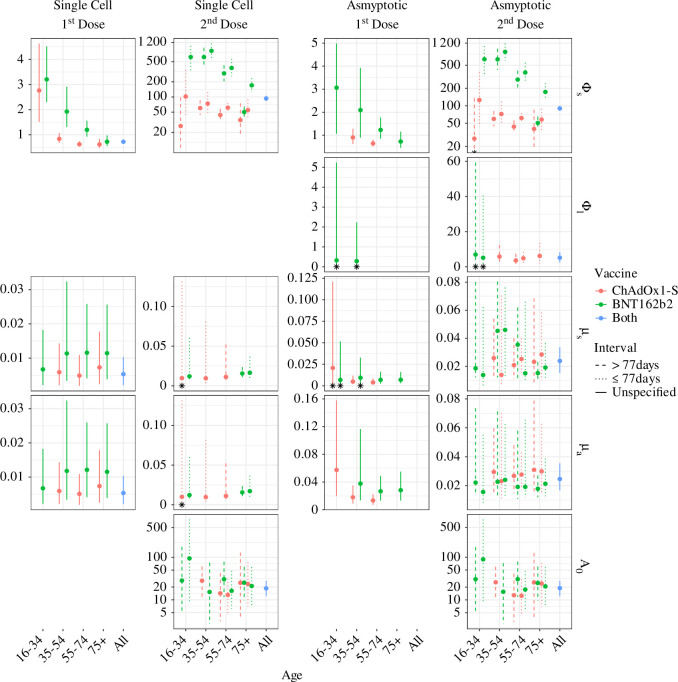
The mode and 95% HDP CrI of the parameter's posterior distribution for both doses and models, when the parameter is identifiable. The line type is used to distinguish between dosing interval, with dashed and dotted lines used for people who received their second dose >77 days or ≤77 days after their first dose respectively, and solid lines are used for groups without a specified dose interval. A dose interval is not specified for first dose groups, and when we do not split the data for the dose, colours are used to distinguish between the vaccines. For the second dose, only one vaccine is specified for the groups because we only have adequate data for homologously vaccinated people (e.g. someone who received ChAdOx1-S for their second dose also received it for their first dose). Red is used for ChAdOx1-S, green for BNT162b2, and blue for when we do not split the data by vaccine brand. Blue points with blue solid error bars represent the estimates and 95% HDP CrI of parameters fit to all the data on that dose. The black asterisks mark when the lower bound of the parameters 95% HDP CrI is zero.

Even though the models can only describe the dynamics of 14 of the 24 groups, information about individual parameters can be extracted from all groups. Figure 3 shows the mode and 95% HPD CrI of the posterior distribution of all identifiable parameters.

A common pattern for both models after both doses is that the value of $\Phi_{s}$ (the antibody production capacity of short-lived plasma cells) decreases with age and is greater for those who received BNT162b2 than those who received ChAdOx1-S. This result explains the higher peak antibody response after vaccination in younger people and those vaccinated with BNT162b2, observed in figure 2. Similarly, comparing the estimates of $\Phi_{s}$ between the first and second doses, we see that both models estimate an increase of 10- to 100-fold and 70- to 440-fold after the second dose of ChAdOx1-S and BNT162b2, respectively. These increases explain the higher peaks after two doses observed in figure 2.

A weak distinction between the group estimates of $\mu_{s}$ and $\mu_{a}$ (short-lived plasma cell decay rate and antibody decay rate, respectively) in the first dose groups is found by both models. The model fitting suggests people 35 and older who received BNT162b2 have faster rates of plasma cell and antibody decay than those who received ChAdOx1-S. This explains why people vaccinated with one dose of ChAdOx1-S maintain a higher antibody level than the same age group who received one dose of BNT162b2, as observed in figure 2. However, this pattern is not found after the second dose.

Under the assumptions about plasma cell production and decay of this model, for there to be evidence of a long-term antibody response in a group, $\Phi_{l}$ (the antibody production capacity of long-lived plasma cells) must be greater than zero. Only people 35 and older who received two doses of ChAdOx1-S were found to show strong evidence of a long-term antibody response. For 35−54 year-olds, this is only true if they received their doses more than 77 days apart, and for people 75 and older, this is only true if they received their doses less than 77 days apart. The data collected from 16–34 years-olds and 35–54 year-olds vaccinated with one dose of BNT162b2, and 16–34 year-olds vaccinated with two doses of BNT162b2 was able to identify $\Phi_{l}$, suggesting these groups could have mounted a long-lived immune response. However, the 95% HDP CrI are [0−5.24], [0−2.23], [0−59.4], and [0−40.6] respectively, all include zero, and therefore we cannot be confident long-lived plasma cells are induced.

Taken together, these observations provide quantifiable explanations for observed differences in antibody dynamics between vaccine types and age groups. They show that we only find evidence of a persisting antibody response and thus likely induction of long-lived plasma cells in people vaccinated with two doses of ChAdOx1-S. They also explain greater peak antibody response observed in younger people than older people, people who receive one dose than people who receive two, and people vaccinated with BNT162b2 than people vaccinated with ChAdOx1-S, by more productive short-lived plasma cells (plasmablast).

## 4. Discussion

We have proposed and analysed two mechanistic and identifiable models of SARS-CoV-2-specific antibody dynamics that link cross-sectional seroprevalence data to within-host antibody dynamics. Our models relate peak antibody levels to the productive capacity of short-lived plasma cells, explaining the observation that individuals who received BNT162b2 have higher peak levels than those that received ChAdOx1-S [7], as well as a higher peak observed in younger people than older people [1–4]. Persistent antibody levels observed in people who receive two doses of ChAdOx1-S are presumed to be explained by the induction of long-lived plasma cells. We found no effect of age on the apparent production capacity of long-lived plasma cells. The inability of mRNA COVID-19 vaccines to elicit long-lived plasma cells after one and two doses has been discussed previously [54]. However, long-lived plasma cells in bone marrow, and improved immunogenicity, have been found in people vaccinated with three and four doses of mRNA COVID-19 vaccines [55,56], although this may be due to infection.

Direct comparison between the parameter estimates of exponential decay models and the models proposed here is difficult as they do not measure the same quantities. The decay rate in exponential models measures the decay in antibody levels and therefore, incorporates the effects of antibody production and degradation. Such approaches are appropriate for assessing the overall decay of antibody levels, especially when data are concentrated in the decay phase [57]. Whereas our mechanistic models separate the processes, with our point estimates of antibody half-life (23–116 days) being consistent with *in vitro* experimental findings [35,47,49–52] and some previous estimates [15,16]. Further, estimates of antibody degradation from more complex mechanistic models, fit to data on multiple immune factors, lie within the 95% CrI intervals of our estimates [19]. Similarly, our estimates of short-lived plasma cell decay rates after the second dose are consistent with estimates from average values in experimental data (0.0389) [58]. However, after the first dose, our model overestimates plasma cell lifespan. Further, our estimates of short-lived plasma cell decay rates are comparable to but smaller than those of other mechanistic models [18,19]. This suggests a lack of data further out from the first dose and may be an effect of excluding plasma cell production ensures parameter identifiability when only antibodies are observed. Instead, it is assumed that the initial population of plasma cells is the full population that decays over time. A consequence of this modelling structure is that we cannot include the expansion of plasma cell populations or other mechanisms of antibody maintenance that involve continued plasma production. More accurate models include the initial expansion of the plasma cell population. Such models fit the initial antibody response better [19] and could give information on differences in plasma cell generation post-vaccination between population groups. Fitting such a model to the data used here would be most improved for the early phase of the first dose response, as this is when we would not expect people to have SARS-CoV-2 specific plasma cells prior to vaccination. However, to be structurally identifiable, such models require data on difficult-to-measure immune factors such as germinal centre B cells and can struggle with practical identifiability due to their complexity [18,19].

Another limitation is the length of time over which the data were collected. Data that continued further after each dose would help clarify long-term dynamics and help validate our predictions. Further, splitting the data potentially removes collective information about true means and parameters that are shared across groups, especially at later time points. Hierarchical fitting could resolve this issue. However, hierarchical modelling enforces a relationship between the parameters of the groups, requiring additional assumptions that could impact comparisons between the groups. These data also do not contain repeated samples. Hence, we cannot account for individual trajectories within the groups and rely on the assumption that individuals within a group are representative of each other. Another limitation is the models cannot distinguish between plasma cell number and rate of antibody production, due to identifiability issues. So, we cannot determine which combination of these two factors is responsible for variation in the overall production of antibodies between groups.

Our models suggest that while mRNA vaccines induce larger short-term responses, which may have advantages for rapid protection, the adenoviral vector vaccines may have advantages in eliciting persistent serum antibodies against SARS-CoV-2, presumably through induction of long-lived plasma cells. Vaccinating people who have only received mRNA vaccines with adenoviral vector vaccines, with priority given to older people, may achieve persistently high antibodies in all age groups. Clinical studies into the effectiveness of BNT162b2 and ChAdOx1-S against infection with SARS-CoV-2 and hospitalization with COVID-19 find that BNT162b2 and ChAdOx1-S are comparable after one dose, but BNT162b2 is more effective after two doses [59–65]. However, comparisons of BNT162b2 and ChAdOx1-S vaccine effectiveness are usually short term, at most up to 30 weeks, whereas our models cover longer timescales. Further, we cannot be certain whether heterologous vaccination will elicit the same humoral response and effectiveness as homologous vaccination. Clinical studies have found that heterologous vaccination is more effective than homologous vaccination at protecting against infection [66–69]. However, these findings are for adenoviral vector-primed mRNA-boosted individuals while we are discussing boosting mRNA-primed individuals with adenoviral vector vaccine. It has been found that binding and neutralizing antibodies wane less in individuals primed with mRNA vaccine and boosted with adenoviral vaccine than in individuals vaccinated with other heterologous vaccination regimens [70,71], supporting mRNA priming and adenoviral vector boosting to achieve persistently high antibody levels.

## 5. Conclusion

Routinely collected seroprevalence data are valuable for characterizing within-host mechanisms of antibody production and persistence. Extended sampling and linking seroprevalence data to outcomes would allow for powerful conclusions on the relationship between humoral kinetics and protection against disease. With added data on other immune factors, such as serum memory B cells and plasmablasts, mathematical modelling can give insights into the complex kinetics of the humoral response. Closer interaction between mathematical modellers and immunologists on data collection and model structure will equip interdisciplinary groups with the necessary information to describe population immune responses more accurately, benefiting the fields of immunology and epidemiology. With the current data, we can make projections of antibody responses into the future, and our results suggest older people who have not received adenoviral vector vaccines may derive longer-lasting protection from a booster dose of this vaccine type than from additional mRNA vaccination.

## Data Availability

The United Kingdom Health and Security Agency (UKHSA) dataset can be accessed by researchers; approval is on a project-by-project basis (https://www.rcgp.org.uk/representing-you/research-at-rcgp/research-surveillance-centre/supporting-research-teams). Supplementary material available online [72].

## Appendix A. Deriving the asymptotic and single-cell models

The general model proposed in [21] is the ‘*complete*’ exponential model that captures the kinetics of short-lived plasma cells $P_{s}$, long-lived plasma cells $P_{l}$, and antibodies A:

(A 1a)
$$\begin{array}{rl} \frac{dP_{s}}{dt} & =-\mu_{s}P_{s}, \end{array}$$

(A 1b)
$$\begin{array}{rl} \frac{dP_{l}}{dt} & =-\mu_{l}P_{l}, \end{array}$$

(A 1c)
$$\begin{array}{rl} \frac{dA}{dt} & =\phi_{s}P_{s}+\phi_{l}P_{l}-\mu_{a}A, \end{array}$$

where $\mu_{s}$, $\mu_{l}$ and $\mu_{a}$ denote the decay rate of short-lived plasma cells, long-lived plasma cells and antibodies respectively, and $\phi_{s}$ and $\phi_{l}$ denote the rate short- and long-lived plasma cells secrete antibodies, respectively. However, it was found in [21] that assuming $\mu_{l}=0$ best describes longitudinal data on hepatitis A-specific antibodies with samples taken up to 114 months after vaccination. Hence, we set $\mu_{l}=0$, implicitly assuming long-lived plasma cells live forever, and solve the system of equation (A 1) with the initial conditions $P_{s}(0)=P_{s}^{0}\text{, }P_{l}(0)=P_{l}^{0}\text{, and }A(0)=A_{0}$ to obtain,

(A 2)
$$\begin{array}{lcr} & A(t)=\frac{\Phi_{l}}{\mu_{a}}+\frac{\Phi_{s}}{\mu_{a}-\mu_{s}}e^{-\mu_{s}t}+\left(A_{0}-\frac{\Phi_{l}}{\mu_{a}}-\frac{\Phi_{s}}{\mu_{a}-\mu_{s}}\right)e^{-\mu_{a}t}, & \end{array}$$

where $\Phi_{s}=\phi_{s}P_{s}^{0}$ and $\Phi_{l}=\phi_{l}P_{l}^{0}$. The new parameters $\Phi_{s}$ and $\Phi_{l}$ represent the maximum antibody production of the respective plasma cell populations and are defined so that equation (A 2) is structurally identifiable when only antibodies are observed. We will refer to $\Phi_{s}$ and $\Phi_{l}$ as the productive capacity of short-lived and long-lived plasma cells, respectively.

We also consider a related model (the single-cell model) that assumes long-lived plasma cells are not induced ($\Phi_{l}=0$).

(A 3)
$$\begin{array}{lcr} & A(t)=\frac{\Phi_{s}}{\mu_{a}-\mu_{s}}\left(e^{-\mu_{s}t}-e^{-\mu_{a}t}\right)+A_{0}e^{-\mu_{a}t}. & \end{array}$$

## Appendix B. Parameter identifiability

We use the STRIKE GOLDD Matlab package [73] (available at https://github.com/afvillaverde/strike-goldd) to determine whether our models are structurally identifiable. We use STRIKE GOLDD because it considers initial conditions as parameters and has tools for reparameterization in the case of structural non-identifiability.

The true value of a structurally identifiable parameter is theoretically possible to find, given noise-free data. It may not be possible to determine the true value of a structurally identifiable parameter when fitting to noisy or sparse data. Practical identifiability, defined as the ability to define finite bounds around an estimate [74], determines whether the data contain enough information to estimate the parameter value with confidence.

An established method of defining confidence intervals is with the profile likelihood method [74], by assuming values of the parameter and maximizing the likelihood function with respect to the remaining parameters (the *‘nuisance’* parameters) [74–76]. The simplest implementation of maximization uses maximum likelihood estimation (MLE) and gradient descent methods [74,76]. Though the methodology of the profile likelihood method is well defined, it can be fragile when likelihood spaces are complex and difficult to navigate by gradient descent.

To mitigate the fragility of the likelihood profile method, we use a Markov chain Monte Carlo (MCMC) algorithm to explore the parameter space more completely and generate posterior distributions of parameter values. We determine a parameter to be practically identifiable if the 95% highest posterior density credible interval (HPD CrI) of the posterior distribution is constrained to a single biologically meaningful (entirely positive) set and is not sensitive to prior assumptions. We say the parameter is not identifiable when the 95% HPD CrI is made up of multiple separate regions because this implies the data do not contain enough information to constrain the parameter estimate around the true value. Similarly, if the 95% HPD CrI is sensitive to prior assumptions, this implies the data do not contain enough information to inform the parameter.

Though multimodal distributions could represent varying immune responses within the group, they are not found in groups with large sample sizes (see electronic supplementary material). This supports the assumption that multimodal distributions are caused by a lack of information within the data, rather than varying immune responses.
